# Case Report: Pembrolizumab associated lichen planus in early stage triple negative breast cancer

**DOI:** 10.3389/fonc.2025.1726097

**Published:** 2026-01-07

**Authors:** Ozge Buyukahisha, Abdullah Ozcelik, Deniz Baycelebi, Asena Cakir, Bulent Cetin

**Affiliations:** 1 OMU Medicine Faculty of Medical Oncology Department, Samsun, Türkiye; 2 OMU Medicine Faculty of Pathology Department, Samsun, Türkiye; 3 OMU Medicine Faculty of Dermatology Department, Samsun, Türkiye

**Keywords:** breast cancer, immune-related side effects, immunotherapy, lichen planus, pembrolizumab

## Abstract

Triple negative breast cancer (TNBC) is a breast cancer with a poor prognosis, marked by the absence of estrogen (ER) and progesterone (PR) receptors as well as human epidermal growth factor receptor (HER2) expression. Although TNBC is characterized by a high recurrence rate and poorer survival, it is more sensitive to chemotherapy compared to other breast cancer subtypes. The use of targeted immunotherapy approaches has been brought to the agenda for the treatment of both early and advanced TNBC, as TNBCs are immunogenic active tumors. In systemic therapy, addition of immune checkpoint inhibitors to cytotoxic chemotherapy is used as part of the neoadjuvant treatment approach. Pembrolizumab is a monoclonal antibody that blocks the interaction between the programmed death-ligand 1 (PD-L1) receptor on T cells and the PD-L1 and PD-L2 ligands on tumor cells. It has been shown to be effective in TNBC, melanoma, lung cancer and other advanced solid tumors and hematologic malignancies. Several skin-related side effects have been documented, such as pruritus, maculopapular rashes, vitiligo, lichenoid skin reactions, psoriasis, and, in rare cases, severe and potentially life-threatening conditions. We report a rare case of pembrolizumab-associated lichen planus in a 43-year-old woman who received pembrolizumab for neoadjuvant treatment of TNBC.

## Introduction

Triple-negative breast cancer (TNBC) accounts for approximately 10–15% of all breast cancers and is defined by the absence of estrogen receptor, progesterone receptor, and HER2 expression. It is associated with aggressive clinical behavior, early relapse, and limited treatment options (1). Although conventional chemotherapy remains the backbone of treatment, outcomes have historically been suboptimal.

The integration of immune checkpoint inhibitors (ICIs), particularly pembrolizumab, into neoadjuvant chemotherapy has transformed the therapeutic landscape of early-stage TNBC. The KEYNOTE-522 trial demonstrated that adding pembrolizumab to standard chemotherapy significantly improved pathologic complete response and event-free survival, establishing ICIs as a key component of treatment in this setting (1).

With the expanding use of pembrolizumab in earlier stages of breast cancer, the recognition and management of immune-related adverse events (irAEs) have gained increasing clinical importance. Cutaneous irAEs are among the most common toxicities associated with ICIs, occurring in up to 40% of treated patients (2–4). These reactions range from mild pruritus and maculopapular eruptions to more characteristic immune-mediated dermatoses such as vitiligo, psoriasis, and lichenoid dermatitis (2–5).

Lichenoid eruptions, which clinically and histopathologically resemble idiopathic lichen planus, have been well documented in association with anti–PD-1/PD-L1 therapies. These reactions are most frequently reported in patients receiving ICIs for melanoma or lung cancer, typically developing after several cycles of therapy and generally responding to topical or systemic corticosteroids (2, 5, 6). However, cases occurring in the neoadjuvant setting for early-stage TNBC are exceedingly rare, and to our knowledge, no prior biopsy-confirmed pembrolizumab-associated lichen planus has been described in this specific context.

Because early-stage TNBC is treated with curative intent, irAEs that interrupt therapy have different clinical implications compared with metastatic disease. Therefore, the early recognition of dermatologic irAEs, prompt initiation of appropriate dermatologic management, and careful multidisciplinary decision-making are crucial to maintain oncologic efficacy while minimizing morbidity (7–9).

This case contributes to the expanding understanding of pembrolizumab-associated cutaneous irAEs by presenting a rare instance of lichen planus developing during neoadjuvant therapy for early-stage TNBC. It underscores the importance of vigilance for atypical dermatologic toxicities and highlights the need for personalized management approaches to balance toxicity control with optimal cancer outcomes.

## Case report

A 43-year-old premenopausal Asian woman was presented to our hospital with a painless palpated mass in the left breast in March 2024. The patient is non-smoker and non-alcoholic. There is no history of malignancy in the patient’s family history, not even breast cancer. The patient mentioned noticing the mass two months prior to her presentation during self-physical examination, with no other symptoms and no previous radiological examinations. On presentation, physical examination revealed a painless palpable mass with well-defined borders and good mobility, approximately 4 cm in diameter, in the upper middle quadrant of the left breast with no skin changes, and her laboratory findings were within normal limits. Mammography revealed a mass measuring approximately 4 cm in the upper middle quadrant of the left breast, with no definite lesion in the contralateral breast or axilla. Preoperative ultrasonography showed a hypoechoic lesion in the upper middle quadrant of the left breast, measuring about 4 × 3.3 cm, with clear borders, regular morphology, and increased blood flow signal (BI-RADS 4). The clinical stage was cT2N0M0. After core needle biopsy, she was diagnosed with invasive ductal breast carcinoma. Immunohistochemical results indicated that the tumor was negative for the estrogen receptor, progesterone receptor and human epidermal growth factor receptor 2 (HER2) expression, with a Ki-67 score of 40% suggesting TNBC. No distant metastases were detected in radiological examinations, including brain, chest, and abdominal scans and BRCA 1–2 test result was evaluated as negative. As a result of discussions with the patient, neoadjuvant chemotherapy (NAC) was recommended for treatment. She was started on neoadjuvant therapy consisting of weekly paclitaxel 160 mg and carboplatin AUC 2 (approximately 250 mg weekly) for 12 weeks, combined with pembrolizumab 200 mg administered every 3 weeks. This was followed by dose-dense doxorubicin 120 mg and cyclophosphamide 1200 mg every 2 weeks, according to a modified KEYNOTE-522 regimen.

**Figure 1:**
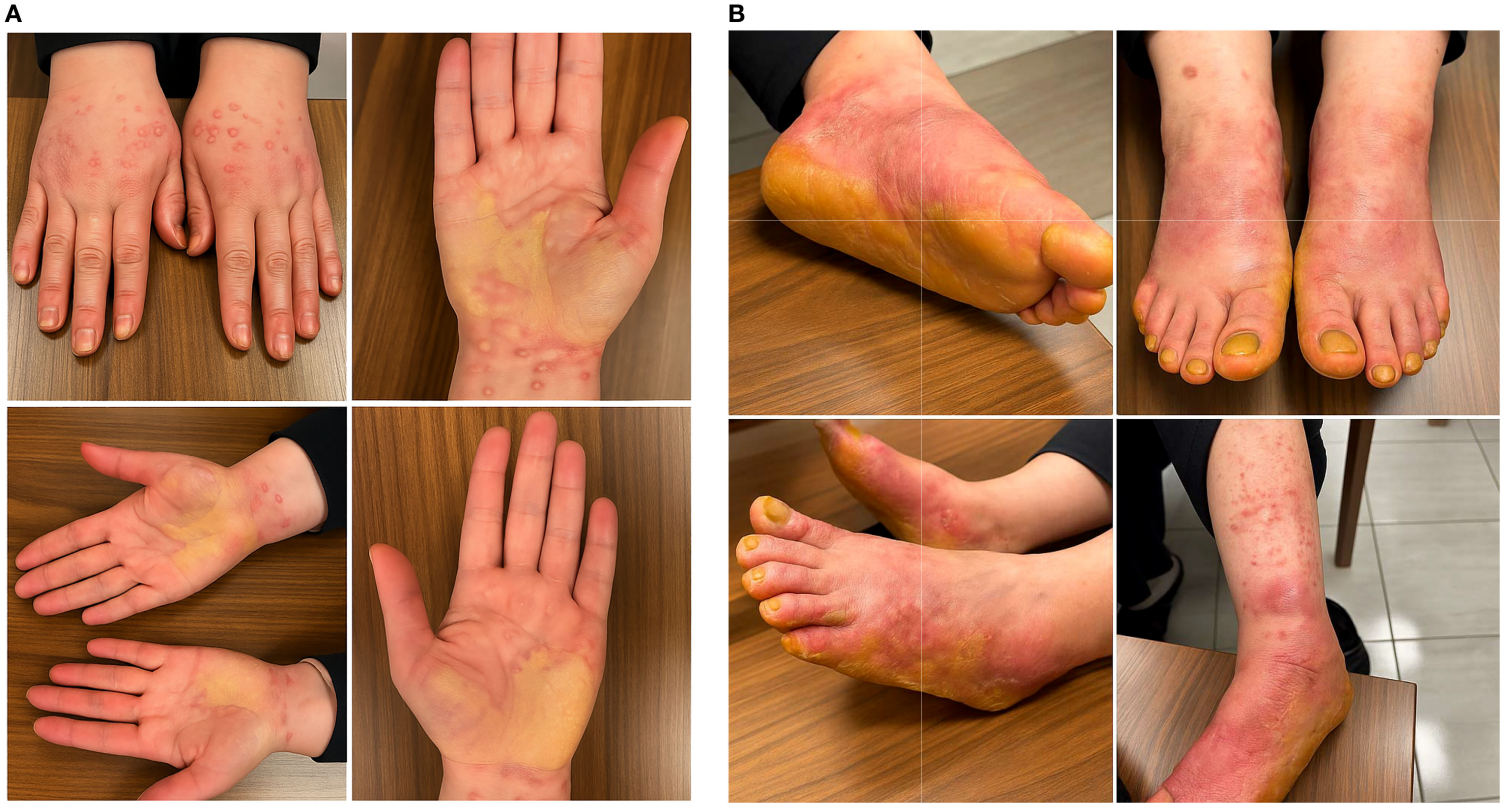
**(A)** Multiple, clinically similar, erythematous to violaceous, firm pruritic papules and plaques on both upper extremities, with acral-predominant distribution. **(B)** Multiple, clinically similar, erythematous to violaceous, firm pruritic papules and plaques on both lower extremities, with acral-predominant distribution.

**Figure 2:**
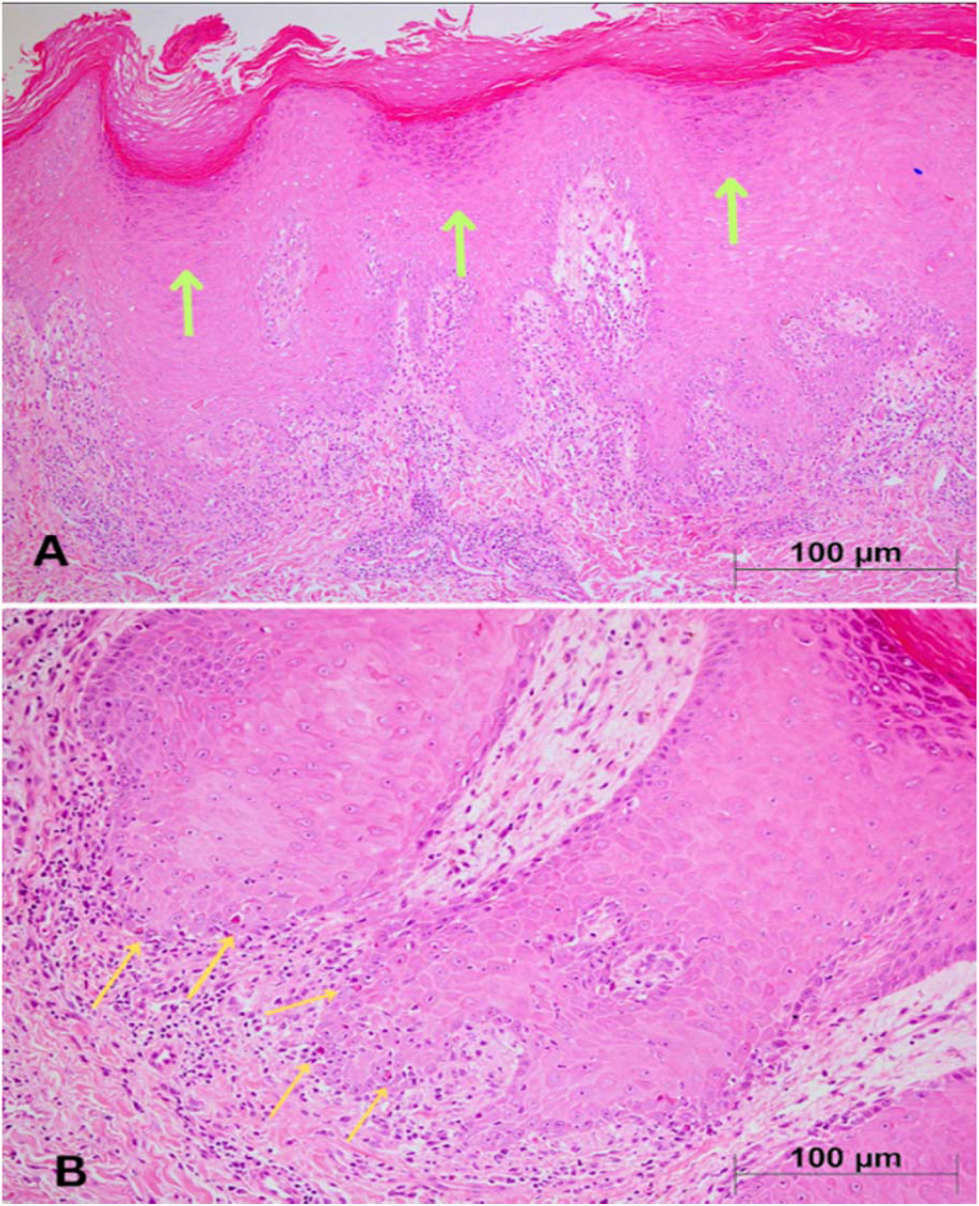
**(A)** Hyperkeratosis and wedge-shaped hypergranulosis (green arrows) are seen in the epidermis. The epidermis also shows sawtooth acanthosis and dermoepidermal junction infiltrated by lymphocytes (H&E, 100X). **(B)** At the higher magnification, necrotic kerotinocytes are seen at the base of the epidermis (yellow arrows) (H&E, 200X).

**Figure 3:**
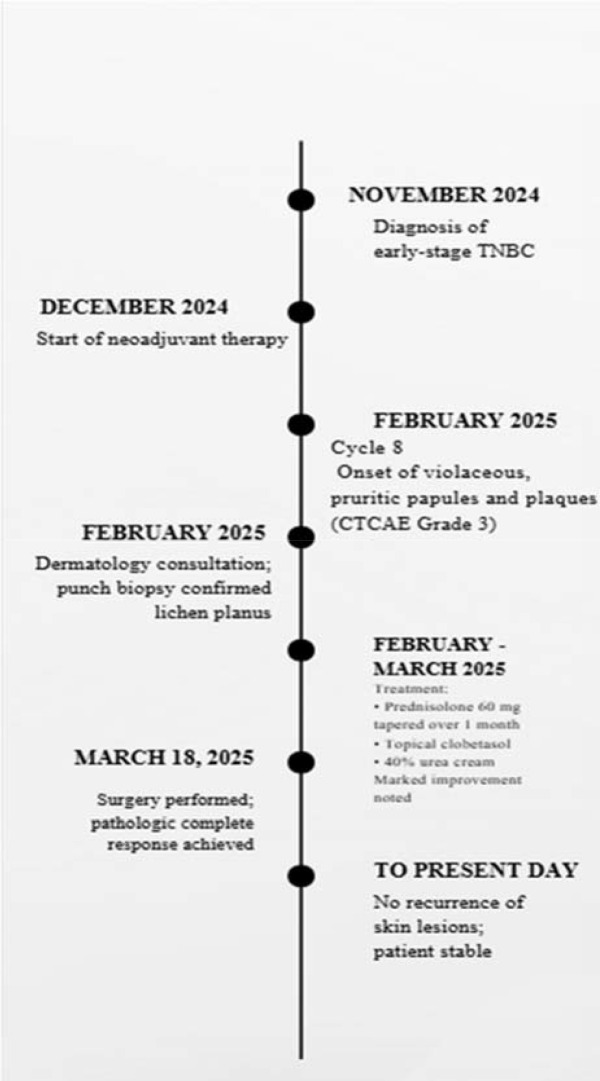
Clinical timeline.

The patient tolerated the first cycles well. After receiving her 8th dose of pembrolizumab (approximately week 24 of therapy), she developed violaceous, pruritic papules and plaques primarily in both upper and lower extremities, accompanied by pain and functional limitation in gripping and walking (**Figure 1**). The estimated affected Body Surface Area (BSA) was 5–10%, and the reaction met the criteria for CTCAE Grade 3 immune-related cutaneous toxicity due to functional impairment and need for systemic corticosteroids. The patient’s baseline laboratory evaluation, including complete blood count, liver and renal function tests, and inflammatory markers, was unremarkable. Serologic testing for viral hepatitis (HBV, HCV) and HIV was negative. Histopathological examination of the biopsy specimen revealed hyperkeratosis on the surface, wedge-shaped hypergranulosis in the epidermis, and sawtooth acanthosis in some areas. Intensified lymphocytic infiltration and necrotic keratinocytes were seen at the dermoepidermal junction. These findings were interpreted as supporting lichen planus (**Figure 2**). A biopsy performed by a dermatologist confirmed lichen planus and was thought to be related to pembrolizumab immunotherapy. The patient was treated with 0.05% topical clobetasol propionate ointment applied twice daily to the hands and feet, and oral prednisone 60 mg daily (methylprednisolone equivalent), tapered by 5 mg every 5 days. The total duration of systemic corticosteroid therapy was approximately 4 weeks. Additionally, 40% urea cream was used twice daily. Her condition improved without any sequelae during the initial 3-month dermatologic follow-up (**Figure 3**).

Due to the CTCAE Grade 3 cutaneous irAE, pembrolizumab was permanently discontinued after Cycle 8 and was not resumed in the adjuvant phase. The patient underwent surgery on March 18, 2025, and achieved a pathologic complete response.

### Diagnostic assessment

Following the onset of violaceous, pruritic papules and plaques on the patient’s extremities, a comprehensive diagnostic evaluation was conducted to determine the etiology and to distinguish between potential immune-mediated or drug-related dermatoses.

The clinical differential diagnosis included:

▪ idiopathic lichen planus,
▪immune checkpoint inhibitor (ICI)-induced lichenoid eruption,
▪ lichen planus pemphigoides,
▪ bullous pemphigoid,
▪ chronic eczematous dermatitis,
▪ psoriasis,
▪ drug-induced hypersensitivity reactions.

Baseline laboratory tests, including complete blood count, liver and renal function tests, ESR, CRP, and thyroid function, were within normal limits. Viral serologies (HBV, HCV) were obtained to rule out hepatitis-associated lichen planus and were negative.

Although testing for bullous pemphigoid (BP180/BP230) autoantibodies is recommended in the evaluation of lichenoid eruptions with possible pemphigoid overlap, this assay was not available in our institution at the time, and therefore could not be performed at that time. However, several clinical and histopathological features made bullous pemphigoid or lichen planus pemphigoides unlikely:

▪ absence of tense bullae,
▪ absence of mucosal involvement,
▪ lack of urticarial plaques or widespread blistering,
▪ absence of subepidermal clefting on biopsy,
▪ absence of eosinophil-rich infiltrates,
▪ and a histologic pattern consistent with classic lichen planus rather than a pemphigoid process (10–12).

A punch biopsy confirmed the diagnosis by demonstrating hallmark features of lichen planus: wedge-shaped hypergranulosis, basal vacuolar degeneration, Civatte bodies, acanthosis, and a dense, band-like lymphocytic infiltrate at the dermoepidermal junction (10, 11). Taken together, clinical presentation and histopathologic findings supported the diagnosis of pembrolizumab-associated lichen planus.

## Discussion

Lichenoid reactions are an increasingly recognized subset of cutaneous immune-related adverse events (irAEs) induced by PD-1/PD-L1 inhibitors. They arise through enhanced autoreactive T-cell activation and loss of peripheral tolerance, leading to cytotoxic injury at the dermoepidermal junction—mechanisms that resemble idiopathic lichen planus but are potentiated by immune checkpoint blockade (4, 10, 12–14). Histopathologically, these reactions are characterized by wedge-shaped hypergranulosis, basal vacuolar degeneration, Civatte bodies, acanthosis, and dense band-like lymphocytic infiltrates, mirroring the classic features observed in our patient (10–12).

Cutaneous irAEs are among the most common toxicities of immune checkpoint inhibitors, occurring in 40–60% of treated patients (2–4, 15–17). While maculopapular rash, pruritus, eczema, psoriasis, vitiligo, and bullous pemphigoid form the majority of dermatologic presentations (11, 15, 18), lichenoid reactions account for a smaller proportion, with an estimated incidence of 1–4% (11, 15, 17). Recent clinical series suggest these reactions may be underdiagnosed due to their variable clinical spectrum and overlap with other dermatoses (13, 19).

Most published cases of pembrolizumab-associated lichen planus or lichenoid dermatitis have been reported in patients with melanoma or non–small-cell lung cancer receiving treatment in the metastatic setting (5, 6, 11, 13, 18, 20, 21). Lesions typically occur after several cycles of therapy, often between weeks 6 and 12, and usually involve the trunk or extremities, with occasional mucosal involvement. Few reports have described acral-dominant presentations, making our case notable, as the patient exhibited widespread acral involvement associated with functional impairment.

### Significance of this case compared with existing literature

Although lichenoid reactions have been documented across various tumor types, our case is unique because it occurred during neoadjuvant pembrolizumab-based chemotherapy for early-stage triple-negative breast cancer (TNBC). To our knowledge, no biopsy-confirmed case of pembrolizumab-associated lichen planus in this specific clinical context has been previously reported. This distinction is clinically important because irAEs occurring in the curative setting have different implications than those observed in metastatic disease. Treatment modifications must be balanced against maintaining oncologic efficacy, underscoring the importance of accurate diagnosis and multidisciplinary management.

### Dermatologic irAEs and this case within the broader spectrum

Cutaneous irAEs encompass a wide spectrum of inflammatory, autoimmune, and blistering dermatoses. Their frequency reflects the heightened immunologic activity provoked by ICIs and their complex interactions with skin-resident lymphocytes (4, 18). Lichenoid eruptions, although relatively rare, hold particular immunologic relevance because they directly reflect T-cell cytotoxicity against keratinocytes. Our patient’s presentation fits well within this mechanistic framework and aligns with established morphologic features described in prior literature (10, 11, 20).

However, the prominent acral involvement observed in this case is rarely emphasized in the existing literature, and contributed to clinically significant pain, pruritus, and impaired hand function. This demonstrates that ICI-related lichenoid reactions may present with variable and occasionally debilitating clinical patterns.

### Immunologic considerations and relationship between irAEs and treatment response

Emerging data suggest that irAEs may correlate with enhanced antitumor immune activation and improved oncologic outcomes. Several studies have shown associations between irAEs—especially dermatologic ones—and improved survival in melanoma and NSCLC (17, 22–25, 37). These associations are thought to reflect robust systemic immune activation. Although the relationship between irAEs and response has not been well studied in early-stage TNBC, our patient achieved a pathologic complete response (pCR) despite requiring pembrolizumab discontinuation in the adjuvant period, supporting the hypothesis that irAEs may serve as indirect biomarkers of immune activation.

### Diagnostic rigor and differential diagnosis

The differential diagnosis for lichenoid eruptions includes idiopathic lichen planus, ICI-induced lichenoid dermatitis, lichen planus pemphigoides, bullous pemphigoid, psoriasis, chronic eczema, and drug-related hypersensitivity reactions (4, 10–12, 18). Although BP180/230 autoantibody testing was not available at our institution, several clinical and histopathologic findings made bullous pemphigoid or lichen planus pemphigoides unlikely: absence of tense bullae, lack of mucosal involvement, no subepidermal clefting, absence of eosinophil-rich infiltrates, and a biopsy consistent with classic lichen planus. These findings, combined with the temporal association with pembrolizumab, strongly supported the diagnosis of ICI-induced lichen planus.

### Therapeutic considerations and comparison with previous cases

Management of ICI-associated lichen planus typically includes high-potency topical corticosteroids for mild disease and systemic corticosteroids for more severe or widespread involvement (3, 7, 9, 15). Most published cases have allowed continuation of pembrolizumab after dermatologic improvement (**Table 1**) (6, 11, 13, 20, 21). In our patient, the degree of acral involvement and functional impact warranted permanent discontinuation during the adjuvant phase, which is consistent with ASCO and ESMO recommendations for grade ≥ 3 cutaneous irAEs (7, 9). Importantly, despite pembrolizumab discontinuation, the patient achieved a pCR, indicating that adequate oncologic outcomes may still be attainable even when treatment modifications are required.

**Table 1:** Literature review for reports on patients with pembrolizumab associated lichen planus.

Cases	Age/sex	Type	Stage	Cutaneous presentation irAE	Site	Time to irAE from ICI onset	irAE treatment	Outcome	References
1	82/M	RCC	IV	LPP	Oral mucosa	After first cycle	Oral diphenyl sulfone, cyclosporine gargle, and topical betamethasone	Discontinuation of the pembrolizumab	Maeda et al., 2024 (26)
2	51/F	Malign melanoma	IV	Lichen planus	Upper and lower extremities	After 27 weeks	Acitretin with topical mometasone furoate	NA	Poljak, et al., 2023 (27)
3	70/M	Malign melanoma	IV	Lichen planus	Dorsum of the hands and on the buccal mucosa	After 21 weeks	Topical betamethasone dipropionate	After complete regression of skin lesions, pembrolizumab was continued to 13 months	Poljak, et al., 2023 (27)
4	67/M	NSCLC	IV	Lichen planus	Hands and scalp	After 18 weeks	Topical corticosteroids	NA	Yamashita et al., 2021 (28)
5	72/M	Unknown Primary	IV	Lichenoid dermatitis	Chest, gluteal region, and both upper and lower extremities	After 6 weeks	Oral prednisolone and urea cream 40%	NA	Sethi et al., 2021 (29)
6	66/M	NSCLC	IV	Lichen planus	Lower extremities, lower abdomen	After 19 months	Topical corticosteroids and antihistamine drugs	After 12 weeks pembrolizumab was continued	Sehbai et al., 2022 (30)
7	67/F	NSCLC	IV	Lichen planus	Upper back and right lower leg keratoacanthoma	After first cycle	Topical corticosteroids and intralesional injections of methotrexate	NA	Tembunde et al., 2023 (31)
8	46/F	Adreno-cortical carcinoma	IV	Lichen planus	Trunk and extremities	After 12 weeks	Topical and oral steroids	Discontinuation of the pembrolizumab	Madan et al., 2023 (32)
9	68/F	Pancreatic adeno-carcinoma	NA	Lichen planus	Chest, back, arms, hands, and legs	NA	Acitretin, topical prednisone and Dupilumab	After complete regression of skin lesions, pembrolizumab was continued	Park et al., 2023 (33)
10	77/M	RCC	IV	Lichenoid dermatitis	Flanks, abdomen, thighs, and buttocks	NA	Topical halobetasol, methotrexate 12.5 mg and Dupilumab	After complete regression of skin lesions, pembrolizumab was continued	Park et al., 2023 (33)
11	54/F	Sarcomatoid carcinoma/ head and neck	IV	Lichen planus	Buccal mucosa, lips and on both feet	After 3 months	Topical corticosteroids, oral antihistamines Acitretin	Died to COVID−19 pneumonia 6 months later	Bansal et al., 2023 (34)
12	65/F	Merkel cell carcinoma	IV	, Lichen planus	The dorsal hands, forearms, chest, upper back, lower Extremities	After 21 weeks	Topical and oral steroids	Discontinuation of the pembrolizumab	Kwon et al., 2020 (35)
13	80/F	NSCLC	IV	Lichen planus	Upper and lower extremities, trunk, buttock, back	After 45 weeks	IV corticosteroids	Discontinuation of the pembrolizumab	Wat et al., 2022 (36)
14	63/F	Breast cancer	IV	Bullous tinea diagnosis 3 weeks prior to LPP	Upper and lower extremities, trunk	After 17 weeks	Doxycycline and nicotinamide	Temporarily (resumed after eruption control)	Wat et al., 2022 (36)
15	43/F	Breast cancer	IIA	Pruritic papules and plaques LPP	Upper and lower extremities	After 24 weeks	Topical and oral steroids with urea cream 40%	Discontinuation of the pembrolizumab	Our Case

NA, not available; LPP, lichen planus pemphigoides; RCC, renal cell carcinoma; NSCLC, non-small cell lung carcinoma.

### Clinical implications

This case broadens the clinical understanding of pembrolizumab-associated cutaneous toxicities in early-stage TNBC and highlights several key messages:

▪ Lichenoid reactions can occur early during neoadjuvant therapy, even in patients without prior dermatologic disease.
▪ Acral involvement may be particularly debilitating, requiring systemic treatment and careful functional monitoring.
▪ Treatment discontinuation does not necessarily compromise oncologic outcomes, especially when irAEs may correspond to heightened immune activation.
▪ Multidisciplinary management is critical, particularly in curative-intent settings where treatment decisions directly influence long-term outcomes.

Overall, this case underscores the need for heightened vigilance for atypical dermatologic irAEs in patients receiving ICIs for early-stage TNBC and demonstrates the importance of balancing toxicity management with optimal cancer outcomes.

## Patient perspective

The patient reported that the sudden development of painful, pruritic lesions on her hands and feet caused substantial discomfort and anxiety, particularly because she was receiving neoadjuvant therapy with curative intent. She expressed concern that these symptoms might represent disease progression or a severe treatment-related complication that could jeopardize her cancer therapy. After the diagnosis was clearly explained, she felt reassured and understood that the condition was manageable. She reported significant improvement following corticosteroid treatment and was relieved that her skin symptoms resolved without long-term sequelae. She expressed satisfaction with the multidisciplinary approach and noted that achieving a pathologic complete response strengthened her confidence in the overall treatment plan.

## Conclusions

This case illustrates an uncommon instance of pembrolizumab-associated lichen planus emerging during neoadjuvant therapy for early-stage TNBC, highlighting the need for clinical attentiveness to atypical cutaneous irAEs as immunotherapy becomes embedded in curative breast cancer care. The patient’s pathologic complete response despite discontinuation of adjuvant pembrolizumab lends support to the evolving view that irAEs may reflect meaningful antitumor immune activation. Broader documentation of similar cases will be essential to delineate their prognostic implications and to inform evidence-based management strategies for ICI-related cutaneous toxicity.

## Data Availability

The raw data supporting the conclusions of this article will be made available by the authors, without undue reservation.
